# 1148. Relationship Between the Duration of Symptoms Before and COVID-19 Disease Outcome After Monoclonal Antibody Therapy

**DOI:** 10.1093/ofid/ofac492.986

**Published:** 2022-12-15

**Authors:** Michael J Hubbard, James Polega, Diana K Haggerty, Nicholas A Andersen, Gordana Simeunovic

**Affiliations:** Spectrum Health/Michigan State University College of Human Medicine, Grand Rapids, Michigan; Spectrum Health/Michigan State University, Grand Rapids, Michigan; Spectrum Health, Grand Rapids, Michigan; Spectrum Health, Grand Rapids, Michigan; Spectrum Health, Michigan State College of Human Medicine, Grand Rapids, Michigan

## Abstract

**Background:**

Clinical trials of monoclonal antibodies therapy (MAB) for COVID-19 demonstrated the risk reduction of COVID related hospitalization and death of any cause if administered within the first 7 days from the symptom onset. The Food and Drug Administration (FDA) issued an emergency use authorization (EUA) for MAB within 10 days from the symptom onset. Our objective was to evaluate how duration of symptoms before MAB affects disease outcome following therapy.

**Figure 1:**
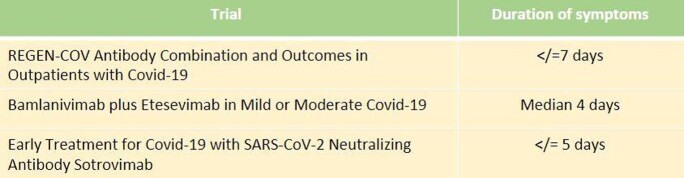
Duration of Symptoms prior to MAB infusion in Clinical Trials

**Methods:**

We evaluated a relationship between symptoms duration prior to MAB and disease outcome following treatment by measuring number of emergency department (ED) visits, hospitalizations and ICU admissions within 14 days and number of deaths within 30 days of MAB. Based on the symptom duration, patients were classified in typical (1-7 days) and late group (8-10 days of symptoms). We evaluated outcomes according to the symptom duration using absolute risk reduction and used Chi-squared tests to assess statistical significance using an alpha of p< 0.05.

**Figure 2:**
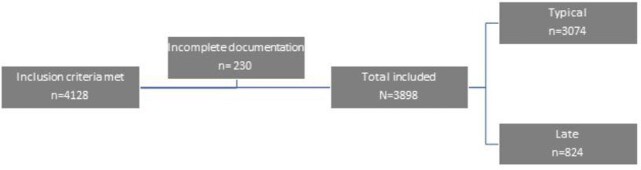
Subject Flow Diagram

**Results:**

From 3898 patients, 3074 (78.9%) were treated within 7 days from the symptom onset. Demographics were similar in both typical and late group. Majority of treated patients in both groups were Non-Hispanic Caucasians suggesting racial and ethnic disparities potentially due to a lack of access to healthcare. All comorbidities were similar or higher in the typical group except for obesity that was more frequent in the late group.

Compared with typical, late group had more ED visits (9.22% vs 7.16% p=0.04) and hospitalizations (4.98% vs 3.68%, p=0.08). Absolute risk of progression to severe disease measured through the number of ICU admissions and deaths was low across the groups, and difference was not statistically significant. Adjusted for demographics and comorbidities, patients from the late group were 1.35 times more likely to seek help in the ED and 1.72 times more likely to get hospitalized.

**Figure 3:**
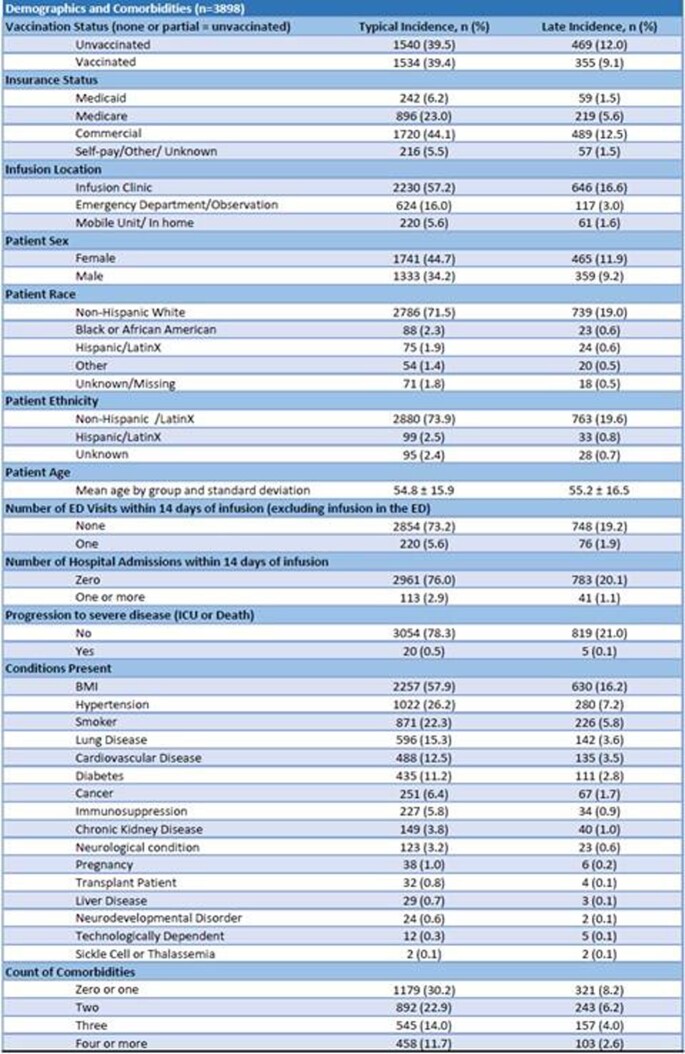
Demographics and comorbidities for Typical (patients treated within 7 days from the symptom onset) and Late (patients treated 8-10 days from the symptom onset) group.

**Conclusion:**

Despite FDA EUA allowing for the use of MAB up to 10 days from the symptom’s onset, our real-world findings suggest that patients benefit most when treatment is administered within 7-day from the symptom onset as consistent with clinical trials.

**Disclosures:**

**All Authors**: No reported disclosures.

**Figure 4:**
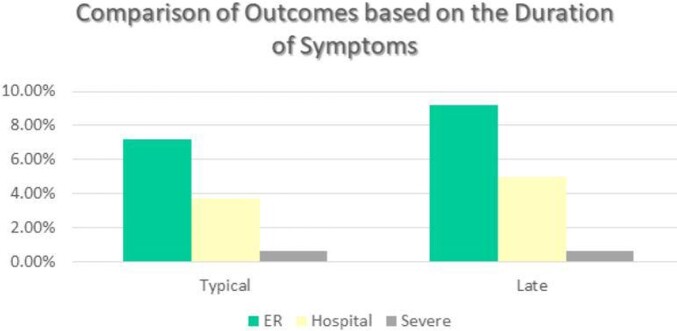
Graphical representation of outcomes related to monoclonal antibody therapy in a typical (1-7 days) duration of symptoms before administration and a late (8-10 days) duration of symptoms before administration.

**Figure 5:**
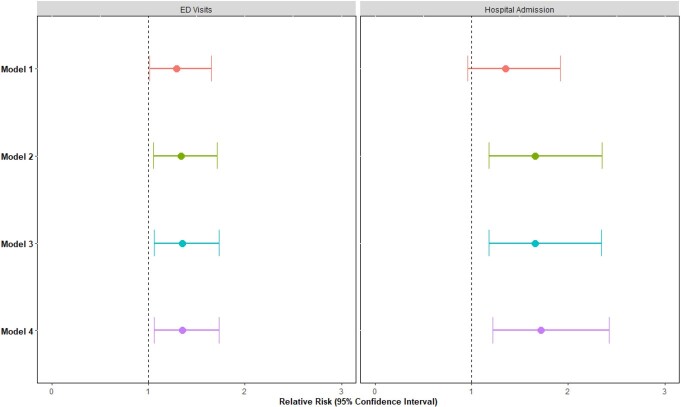
A Forest plot of the models developed to compare the risk reduction (RR) of emergency department (ED) visits and hospital admissions between patients receiving monoclonal antibodies in the typical (1-7 days after symptom onset) timeframe versus the late (8-10 days after symptom onset) timeframe with confidence intervals (CI). Models were adjusted according to the following covariates: Model 1- unadjusted; Model 2 – adjusted for infusion location, patient insurance, sex, age, race, and vaccination status; Model 3 – model 2 variables and the number of comorbidities; and, lastly, model 4 – model 2 variables plus specific comorbidities (diabetes, lung cancer, immunosuppression, body mass index (BMI), chronic kidney disease, cardiovascular disease, lung disease, neurological disorders, and smoking).

